# Mechanisms of tyrosine kinase inhibitor resistance in renal cell carcinoma

**DOI:** 10.20517/cdr.2023.89

**Published:** 2023-12-28

**Authors:** Patrick L. Sweeney, Yash Suri, Arnab Basu, Vadim S. Koshkin, Arpita Desai

**Affiliations:** ^1^Deming Department of Medicine, Tulane University School of Medicine, New Orleans, LA 70112, USA.; ^2^University of Arizona College of Medicine, Tucson, AZ 85724, USA.; ^3^Division of Hematology and Oncology, Department of Medicine, University of Alabama at Birmingham Heersink School of Medicine, Birmingham, AL 35233, USA.; ^4^Division of Hematology and Oncology, Department of Medicine, University of California at San Francisco School of Medicine, San Francisco, CA 94143, USA.

**Keywords:** Antiangiogenic tyrosine kinase inhibitors, renal cell carcinoma, acquired resistance, sunitinib, tumor microenvironment, immune checkpoint inhibitors

## Abstract

Renal cell carcinoma (RCC), the most prevalent type of kidney cancer, is a significant cause of cancer morbidity and mortality worldwide. Antiangiogenic tyrosine kinase inhibitors (TKIs), in combination with immune checkpoint inhibitors (ICIs), are among the first-line treatment options for patients with advanced RCC. These therapies target the vascular endothelial growth factor receptor (VEGFR) tyrosine kinase pathway and other kinases crucial to cancer proliferation, survival, and metastasis. TKIs have yielded substantial improvements in progression-free survival (PFS) and overall survival (OS) for patients with advanced RCC. However, nearly all patients eventually progress on these drugs as resistance develops. This review provides an overview of TKI resistance in RCC and explores different mechanisms of resistance, including upregulation of alternative proangiogenic pathways, epithelial-mesenchymal transition (EMT), decreased intracellular drug concentrations due to efflux pumps and lysosomal sequestration, alterations in the tumor microenvironment including bone marrow-derived cells (BMDCs) and tumor-associated fibroblasts (TAFs), and genetic factors such as single nucleotide polymorphisms (SNPs). A comprehensive understanding of these mechanisms opens the door to the development of innovative therapeutic approaches that can effectively overcome TKI resistance, thereby improving outcomes for patients with advanced RCC.

## INTRODUCTION

Kidney cancer is the 6th most common cancer in men and the 9th most common cancer in women in the United States, and there will be an estimated 82,000 new cases and 15,000 deaths in 2023^[1]^. Renal cell carcinoma (RCC) is the most common type of kidney cancer, accounting for over 90%-95% of all cases^[2]^. With an annual incidence rate of 17.1 per 100,000 individuals and a mortality rate of 3.6%, RCC has become a significant public health concern, resulting in substantial morbidity and mortality^[3]^. RCC originates from renal tubular epithelial cells and comprises a diverse and heterogeneous group of pathologies^[4]^. RCC is categorized into multiple subtypes, the most common of which is clear cell RCC (ccRCC), accounting for approximately 75% of all cases and 85% of metastatic cases^[5,6]^. Other subtypes include papillary, chromophobe, collecting duct, and renal medullary carcinomas. Risk factors for RCC include age, male sex, tobacco use, hypertension, and genetic syndromes such as von Hippel-Lindau (VHL)^[7,8]^.

Management of RCC varies based on disease stage and characteristics. For localized disease, treatment strategies encompass active surveillance, nephrectomy, radiofrequency ablation, and cryotherapy^[9,10]^. Adjuvant systemic therapies including antiangiogenic targeted therapies have thus far failed to demonstrate a benefit in overall survival, although there are several ongoing trials in this space^[11]^. The management of metastatic disease, predominantly ccRCC, is more complicated. Until the mid-2000s, systemic cytokines including interleukin-2 (IL-2) and interferon-alpha (IFN-α) were commonly used, but response rates to these therapies were poor^[12]^. The initial breakthrough in targeted therapy for RCC occurred in 2005 with the approval of sorafenib, a tyrosine kinase inhibitor (TKI) against vascular endothelial growth factor (VEGF). In 2009, bevacizumab, a monoclonal antibody against VEGF, was approved in combination with IFN-α. Subsequently, other multitargeted small molecule antiangiogenic TKIs emerged as treatment options and significantly improved progression-free survival (PFS) and overall survival (OS)^[13]^. Another class of agents, the mammalian target of rapamycin (mTOR) inhibitors, were introduced during the same period. In recent years, the addition of immune checkpoint inhibitors (ICIs) to TKIs in the frontline setting has reshaped the treatment landscape of advanced RCC^[14]^.

**Figure 1:**
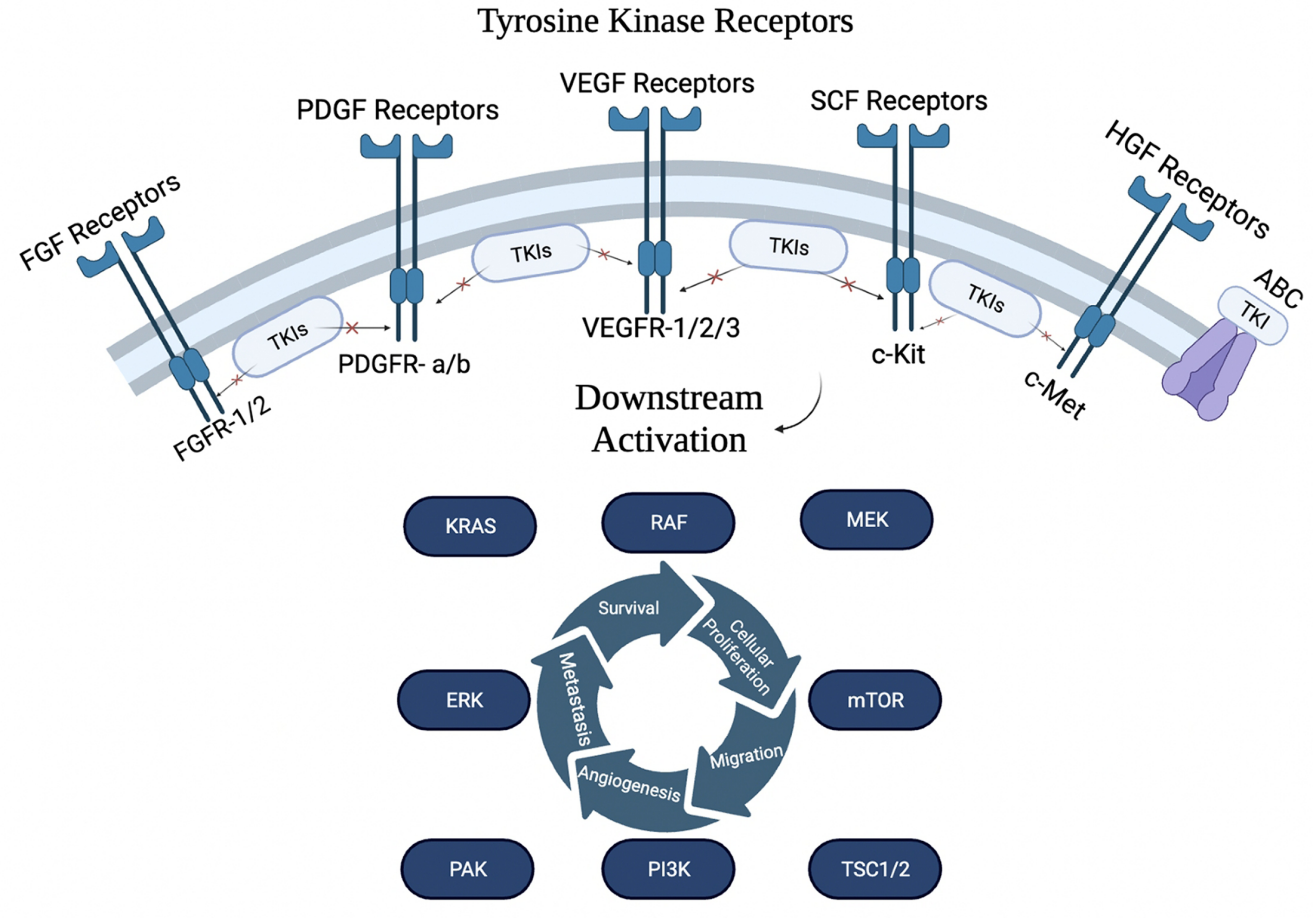
Common receptor tyrosine kinases and their downstream signaling targets. ABC: ATP-binding cassette; ERK: extracellular signal-related kinases; FGF: fibroblast growth factors; FGFR: FGF receptor; HGF: hepatocyte growth factor; mTOR: mammalian target of rapamycin; PDGFR: platelet-derived growth factor receptor; PI3K: phosphoinositide-3-kinase; TKIs: tyrosine kinase inhibitors; VEGF: vascular endothelial growth factor; VEGFR: vascular endothelial growth factor receptor.

TKIs are competitive inhibitors that bind to the kinase domain of their target receptor tyrosine kinase (RTK). RTKs are transmembrane proteins possessing an extracellular ligand-binding domain and an intracellular kinase domain, enabling them to transmit signals across the plasma membrane. Upon ligand binding, RTKs undergo dimerization and autophosphorylation, leading to kinase domain activation. This provides a docking site for proteins, enabling them to direct a cascade of intracellular events that regulate cellular proliferation, differentiation, survival, and migration [Figure 1]^[15]^. Dysregulation of RTK signaling via constitutive autophosphorylation resulting in ligand-independent kinase activity has been implicated in many cancers, making RTKs attractive therapeutic targets^[16]^. In RCC, the signaling pathways initiated by overactive VEGF receptor (VEGFR) lead to enhanced endothelial cell migration, proliferation, permeability, survival, and lymphangiogenesis^[17]^. Antiangiogenic TKIs have been developed to mute this response by binding at or adjacent to the ATP-binding site, preventing autophosphorylation^[18,19]^. These drugs target various kinases, including VEGFR, platelet-derived growth factor receptor (PDGFR), c-Kit, FMS-related receptor tyrosine kinase 3 (FLT-3), and rearranged during transfection tyrosine-protein kinase (RET)^[20]^. The majority of TKIs are multitargeted and have activity against several kinases with varying potency. Importantly, all TKIs commonly used in the treatment of advanced RCC possess activity against VEGFR^[21]^. These include sunitinib, sorafenib, pazopanib, axitinib, cabozantinib, and lenvatinib.

Despite the success of these targeted agents, antiangiogenic TKI resistance is common and poses significant obstacles to achieving durable responses in the treatment of RCC. For instance, the majority of RCC patients who start on sunitinib will develop resistance within 6 months of treatment^[22]^. In this review, we aim to explore the molecular mechanisms underlying TKI resistance in RCC and shed light on potential strategies to overcome this phenomenon. By elucidating the complexities of TKI resistance, we hope to pave the way for the development of novel therapeutic approaches that can improve outcomes for patients with advanced RCC.

## RATIONALE FOR ANTIANGIOGENIC TKIS IN RCC

The treatment of RCC stems from the underlying molecular and genetic alterations that drive tumorigenesis and progression. Almost all hereditary and most sporadic cases of RCC involve mutations leading to loss of tumor suppressor gene function. The most common mutation in ccRCC is the loss of the VHL via loss of chromosome 3p, seen in over 90% of sporadic cases^[23]^. This leads to the loss or impaired function of VHL tumor suppressor protein (pVHL). Normally, pVHL targets the alpha subunits of hypoxia-inducible factor (HIF) for proteasomal degradation through prolyl hydroxylases. In the absence of pVHL, HIF, particularly HIF-2α, accumulates and acts as a transcription factor to trigger the overexpression of hypoxia-inducible genes such as VEGF, platelet-derived growth factor-BB (PDGF-BB), transforming growth factor-alpha (TGF-α), c-Met, cyclin D1, and CXCR4^[24]^. These genes play a crucial role in angiogenesis, cell proliferation, and tumor growth. HIF-2α also upregulates the production of c-Myc, a transcription factor implicated in the progression of various cancers^[25]^. Antiangiogenic TKIs disrupt this aberrant molecular cascade by specifically targeting the dysregulated signaling pathways driven by HIF-2α and its downstream targets, thereby inhibiting tumor angiogenesis and growth in RCC.

## TKI RESISTANCE

Resistance to a targeted agent develops when either the agent is no longer able to inhibit specific signaling pathways or when a tumor’s ability to survive becomes independent from those pathways. Various mechanisms contribute to drug resistance, such as alterations in drug targets, activation of alternative signaling pathways, enhancement of drug efflux, and evasion of apoptotic cell death^[26]^.

Resistance to antiangiogenic targeted therapies can be categorized as intrinsic (primary) or acquired (secondary)^[27]^. Intrinsic resistance is characterized by an inherent insensitivity of cancer cells to TKIs and a lack of response to the drug. By contrast, acquired resistance arises when cancer cells initially respond to TKI treatment but eventually relapse as the drug loses efficacy over time due to the acquisition of various resistance mechanisms. Multiple *in vitro* and *in vivo* models involving cell lines, patient-derived xenografts (PDX), organoids, and genetically engineered mouse models (GEMMs) have been developed to assess the mechanisms, dynamics, and progression of drug resistance^[28]^. These models help elucidate the complex interplay between cancer cells and their microenvironment, offering valuable information for developing strategies to overcome resistance and improve treatment outcomes. In the following section, we summarize the numerous mechanisms of TKI resistance identified to date in RCC.

## ACQUIRED MECHANISMS OF TKI RESISTANCE IN RCC

### Alternative proangiogenic pathways

Successful antiangiogenic TKI therapy suppresses the production of proangiogenic factors such as VEGF and PDGF and inhibits angiogenesis. This eventually leads to tumor hypoxia, which then triggers the upregulation of alternative proangiogenic pathways, contributing to eventual drug resistance. This process is also known as angiogenic escape or angiogenic switch.

One such pathway involves the increased expression of interleukin-6 (IL-6). IL-6 activates the STAT3 pathway, leading to the upregulation of HIF-2α and subsequent increased production of VEGFR^[29]^. IL-8 is also upregulated as a response to hypoxia and contributes to angiogenesis by promoting endothelial cell proliferation, survival, and migration via VEGF mRNA transcription and autocrine VEGFR-2 activation^[30,31]^. High levels of IL-6 and IL-8 have been correlated with significantly shorter PFS and OS in metastatic RCC patients treated with sunitinib and pazopanib^[32,33]^.

Angiopoietin 1 and 2 (Ang 1/2) are critical regulators of angiogenesis, acting as ligands for the Tie2 receptor tyrosine kinase on endothelial cells^[34]^. When Tie2 is activated, it promotes vessel stabilization, survival, and maturation, thereby boosting the VEGF pathway’s effectiveness in improving perfusion to RCC tumors^[35]^. Wang *et al.* followed Ang 2 levels as RCC patients were treated with sunitinib and found that Ang 2 decreased as patients responded to therapy, but then increased as patients became resistant and developed advanced disease^[36]^.

C-Met, a tyrosine kinase encoded by the MET proto-oncogene, binds hepatocyte growth factor (HGF) and initiates an alternative proangiogenic pathway to VEGFR. Type 1 papillary RCC is commonly associated with activating MET alterations and has an unfavorable prognosis^[37]^. Inhibitors against c-Met have emerged as important treatment options for this disease in recent years. In the phase 2 CREATE trial by Schöffski *et al.*, patients with type 1 papillary RCC were categorized by MET mutation status and treated with crizotinib, a TKI against c-Met and ALK. Patients with MET alterations or amplifications were found to have a higher objective response rate, as well as increased PFS and OS compared to patients without MET alterations or amplifications^[38]^. High c-Met expression in ccRCC has been identified as an independent risk factor for higher tumor grade, aggressive phenotype, increased metastasis, and decreased overall survival^[39]^. C-Met activation and overexpression have been identified in ccRCC cells previously treated with anti-VEGFR TKIs, likely conferring resistance to these therapies^[40]^. Increased c-Met expression in patients previously treated with sunitinib has been correlated with shorter PFS and OS^[41]^.

Other proangiogenic factors including fibroblast growth factors 1 and 2 (FGF 1/2) and ephrin A1 and A2 (EFNA 1/2) have also been found to be upregulated following VEGFR inhibition, likely as a result of tumor hypoxia^[42]^. The FGF receptor (FGFR) pathway regulates and activates alternative proangiogenic pathways such as mitogen-activated protein kinases/extracellular signal-related kinases (MAPK/ERK) and phosphoinositide-3-kinase/AK strain transforming signaling pathway (PI3K/Akt)^[43]^.

### Increased pericyte coverage

Pericytes are mural cells that surround the endothelial cells of blood vessels and promote vascular stability and angiogenesis^[44]^. PDGF-BB is secreted by vascular endothelial cells and binds to PDGFR-β on pericytes, activating signaling that leads to increased pericyte production of VEGF which promotes endothelial cell proliferation^[45]^. Increased pericyte activity in the tumor microenvironment has been associated with more aggressive ccRCC^[46]^, and resistance to antiangiogenic TKIs has been observed in tumors with an increased number of pericytes^[47]^. A proposed mechanism of resistance is the inhibition of an important negative feedback loop. High VEGF levels lead to the formation of a VEGFR/PDGFR-β complex that suppresses PDGFR-β signaling, preventing the production of excessive VEGF^[48]^. In the setting of TKI therapy against VEGFR, this mechanism may be less effective, leading to overactive pericyte activity. Thus, all antiangiogenic TKIs induce resistance via this mechanism.

### Multi-drug resistance via lysosomal sequestration and efflux transporters

Sunitib is a small, hydrophobic, weak base, which allows it to easily pass through the cellular membrane, accumulate intracellularly, and bind to the kinase domain of VEGFR^[49]^. However, this structure also lends itself to efficient passage into lysosomes, where a more acidic environment encourages protonation of the drug, conferring a positive charge and effectively sequestering it for degradation^[50]^. Although sunitinib is particularly susceptible due to its chemical properties, lysosomal sequestration has been identified as a contributor to multi-drug resistance (MDR) in cancer cells via a variety of mechanisms. Cancer cells have been found to upregulate PI3K, which promotes lysosomal activity and stability. Further, cancer cells have been found to abolish lysosomal membrane permeabilization (LMP), a process that normally induces apoptosis, via upregulation of cytosolic protease inhibitors and via increased translocation of Hsp70 to the lysosomal lumen, which stabilizes lysosomal membranes^[51]^. Following exposure to sunitinib, RCC cells exhibit a rise in lysosomal mass, facilitating enhanced sequestration^[52]^. Zhitomirsky *et al.* demonstrated that exposure of human carcinoma cells to sunitinib leads to an increase in the number of lysosomes per cell, as well as the number of lysosomes accumulating high levels of sunitinib^[53]^.

Sunitinib exposure has also been associated with increased expression of ATP-binding cassette (ABC) transporters such as P-glycoprotein (P-GP), which are present both on the lysosomal and cell membranes and promote both lysosomal sequestration and extracellular efflux^[54]^. Thus, lysosomal sequestration and efflux transporter proteins work synergistically to promote the development of resistance^[55]^.

### Bone marrow-derived cell recruitment

Bone marrow-derived cells (BMDCs) are a pool of progenitor cells, including hematopoietic stem cells, endothelial progenitor cells, tumor-associated macrophages, mesenchymal stromal cells, VEGFR1-positive hemangiocytes, and more^[56]^. Myeloid-derived suppressor cells (MDSCs) are a subpopulation of BMDCs that most commonly express CD11b and serve an immunomodulatory role in the tumor microenvironment, suppressing the activity of infiltrating cytotoxic T lymphocytes^[57]^. MDSCs produce nitric oxide, which reacts with superoxide, generating peroxynitrite (PNT). PNT nitrates both T-cell receptors, reducing their responsiveness to antigen major histone compatibility (MHC) complexes, and T-cell specific chemokines, blocking T-cell migration^[58]^. MDSCs also produce proangiogenic proteins such as IL-8, MMP8, and MMP9^[59]^. MDSCs and other BMDCs are recruited to RCC tumors by various chemokines, promoting tumor angiogenesis and progression^[60,61]^. Sunitinib has been shown to reduce MDSCs in the peripheral blood of mRCC patients who had the local tumor resected^[62]^. However, persistently high levels of MDSCs have been found in cases of resistant RCC. This may be due to the increased production of granulocyte-macrophage colony-stimulating factor (GM-CSF), which protects MDSCs from sunitinib-induced apoptosis^[59]^. Recruitment of MDSCs and other BMDCs to the tumor microenvironment is likely due to hypoxia induced by TKI therapy. Therefore, though this effect of sunitinib is well-known, all approved antiangiogenic TKIs are thought to contribute to this process.

### Tumor-associated fibroblasts

Tumor-associated fibroblasts (TAFs) are activated fibroblasts that have undergone phenotypic and functional changes in response to tumor-derived signals^[63]^. Unlike regular fibroblasts, TAFs remain chronically activated and continue to carry out their work indefinitely within the tumor microenvironment. They support tumor survival and growth by secreting various growth factors, cytokines, and chemokines, such as interleukins, HGF, and stromal cell-derived factor 1 alpha (SDF-1α)^[64]^. TAFs promote the deposition of a dense extracellular matrix, creating a physical barrier that hampers drug penetration into the tumor and supporting cell adhesion-mediated drug resistance^[65,66]^. Moreover, the role of TAFs in supporting cell adhesion-mediated drug resistance (CAM-DR) is well-defined. TAFs also play a part in reprograming tumor metabolism, making cancer cells less dependent on glucose, and increasing the lactate upload to drive anabolic pathways^[64]^. Platelet-derived growth factor-C (PDGF-C) mediates the angiogenic properties of TAFs and has been found to be upregulated in tumor cells resistant to anti-VEGF antibodies^[67]^.

### Epithelial-mesenchymal transition

Epithelial-mesenchymal transition (EMT) is an embryonic development process that can be hijacked by cancer cells to promote invasion, metastasis, and resistance to therapy. During EMT, epithelial cells lose characteristics such as cell-to-cell adhesion and apical-basal polarity, and acquire mesenchymal features including increased motility, invasiveness, and resistance to cell death^[68]^. This phenotypic switch is achieved by changes in gene expression, such as the downregulation of epithelial markers (E-cadherin) and upregulation of mesenchymal markers (N-cadherin, vimentin), as well as by epigenetic modifications^[68]^. EMT in cancer is largely driven by tumor hypoxia and resultant HIF-1α activation, as well as by inflammatory cytokines IL-6, IL-8, IL-15, and tumor necrosis factor-α (TNF-α)^[20,69]^.

EMT is frequently discussed in the context of cancer stem cells (CSCs), which are subpopulations of cells within tumors that are capable of self-renewal and multi-lineage differentiation^[70]^. Because heterogeneous tumors contain many phenotypically distinct cells with varying degrees of response to chemotherapeutics, CSCs enhance drug resistance and tumor relapse. Processes associated with EMT, such as suppression of E-cadherin, have been shown to generate increased CSCs in tumors^[71]^. A specific side population of CSCs has been identified in RCC, and many RCC tumors express diverse markers associated with CSCs^[72]^.

Acquired resistance to sunitinib via EMT has been demonstrated in patient-derived xenograft models. In a study by Hammers *et al.*, tumor tissue from skin metastases of a sunitinib-resistant ccRCC patient was implanted into nude mice, and these mice were treated with sunitinib or vehicle for 90 days. The average tumor volume was less than 200 mm^3^ in mice treated with sunitinib and more than 800 mm^3^ in mice treated with the vehicle, which suggests renewed sensitivity to sunitinib. The histology of the skin metastases indicated sarcomatous differentiation with a fibroblast-like appearance, indicating EMT. However, the cells in the xenograft returned to normal ccRCC histology^[73]^.

### Pharmacogenomic factors

Single nucleotide polymorphisms (SNPs) are variations in a single nucleotide within the DNA sequence, which achieve an allelic frequency of at least 1% in a population, and they can affect gene expression, protein function, and drug metabolism^[3]^. Specific SNPs in drug-metabolizing enzymes, drug transporters, or drug targets can impact the efficacy of TKIs.

CYP3A4 and CYP3A5 are two of the principal enzymes responsible for sunitinib metabolism, converting the drug to its active metabolite, N-desethyl sunitinib (SU12662)^[74]^. SU12662 has a longer half-life than sunitinib, resulting in increased drug exposure^[75]^. The CYP3A4 SNP rs4646437G>A has been associated with an increased incidence of hypertension in mRCC patients treated with sunitinib. This is presumably due to enhanced sunitinib metabolism leading to increased concentrations of SU12662^[76]^. The expression of CYP3A4 is negatively regulated by two ligand-activated nuclear receptors, NR112 and NR113^[77]^. SNPs present in the genes encoding NR112 and NR113 have been associated with decreased patient PFS and OS, likely due to enhanced NR112 and NR113 activity leading to decreased CYP3A4 expression and lower SU12662 concentrations^[78]^. SNPs in CYP3A5, such as the CYP3A5*1 allele, have been associated with sunitinib toxicity and the need for dose reduction, likely due to enhanced CYP3A5 activity leading to increased concentrations of SU12662^[79]^.

Additionally, SNPs in genes coding for ABC transporters have been found to affect the uptake and efflux of TKIs, altering drug concentrations within tumor cells. The presence of CGT in the ABCB1 haplotype is associated with improved PFS, likely due to decreased clearance of sunitinib and its active metabolite SU12662^[79]^. On the other hand, certain variants such as the TT-genotype in ABCB1 rs1125803 or the TT/TA-variant in ABCB1 rs2032582 have been found to promote drug efflux, resulting in increased intracellular clearance of sunitinib, increased time-to-dose reduction, and decreased PFS^[80]^.

### MicroRNAs

MicroRNAs (miRNAs) are small non-coding RNA molecules that play a significant regulatory role in gene expression. miRNAs are known to contribute to cancer progression by silencing tumor suppressor genes via destruction or decreased expression of mRNA^[81]^. Multiple miRNAs have been implicated in mediating TKI resistance in RCC by targeting key signaling pathways involved in cell proliferation, survival, and drug response. For example, miR-15b has been found to be overexpressed in sunitinib-resistant cell lines after incubation with the drug. *In vivo* models have produced similar results^[82]^. Other notable miRNAs overexpressed in sunitinib-resistant RCC cells include miRNA-575, miRNA-642b-3p, and miRNA-4430 (all studied *in vitro*), as well as miRNA-942, miRNA-133a, miRNA-628-5p, and miRNA-484 (all studied *in vivo*)^[83,84]^. Additionally, miR-144-3p overexpression in ccRCC has been found to enhance cell proliferation, clonogenicity, migration, invasion, and resistance via repression of the tumor suppressor gene, ARID1A^[85]^.

**Figure 2:**
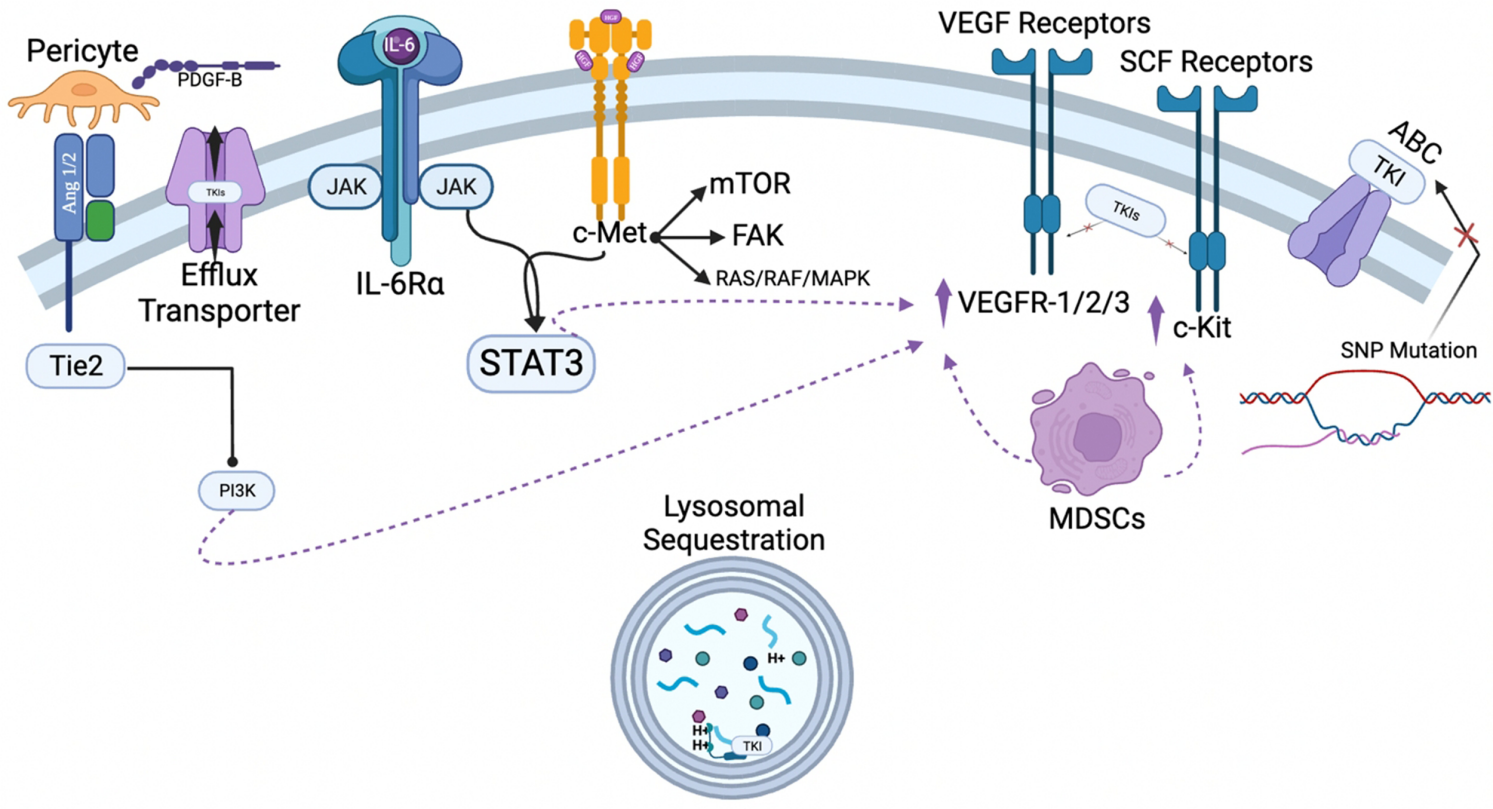
Common acquired mechanisms of resistance to tyrosine kinase inhibitors in RCC. ABC: ATP-binding cassette; IL-6: interleukin-6; MAPK: mitogen-activated protein kinases; MDSCs: myeloid-derived suppressor cells; mTOR: mammalian target of rapamycin; PDGF: platelet-derived growth factor; PI3K: phosphoinositide-3-kinase; RCC: renal cell carcinoma; SNP: single nucleotide polymorphism; TKIs: tyrosine kinase inhibitors; VEGF: vascular endothelial growth factor; VEGFR: vascular endothelial growth factor receptor.

In contrast, miR-200b and miR-141 have been found to be downregulated in ccRCC compared to benign tissue, and their expression may represent an independent prognostic factor for increased PFS and OS^[86]^. Figure 2 illustrates the wide array of known acquired mechanisms of TKI resistance in RCC.

## INTRINSIC MECHANISMS OF TKI RESISTANCE IN RCC

### Methylation of tumor suppressor genes

Enhancer of zeste homolog 2 (EZH2) is an enzyme that functions as the catalytic subunit of the polycomb repressive complex 2 (PRC2). Its primary function is to methylate lysine 27 on histone H3, resulting in gene silencing and transcriptional repression^[87]^. Intrinsic increased expression of EZH2 can lead to aberrant methylation of specific tumor suppressor genes, rendering them inactive. Methylation of these genes can disrupt critical cellular pathways involved in growth control and DNA repair, leading to the promotion of tumorigenesis. The dysregulation of EZH2-mediated methylation can confer resistance to TKIs and other antineoplastic agents by circumventing the inhibitory effects of these drugs on oncogenic signaling pathways. The overexpression of EZH2 has been reported in RCC and is associated with poor prognosis^[88,89]^.

### Inhibition of apoptosis

**Figure 3:**
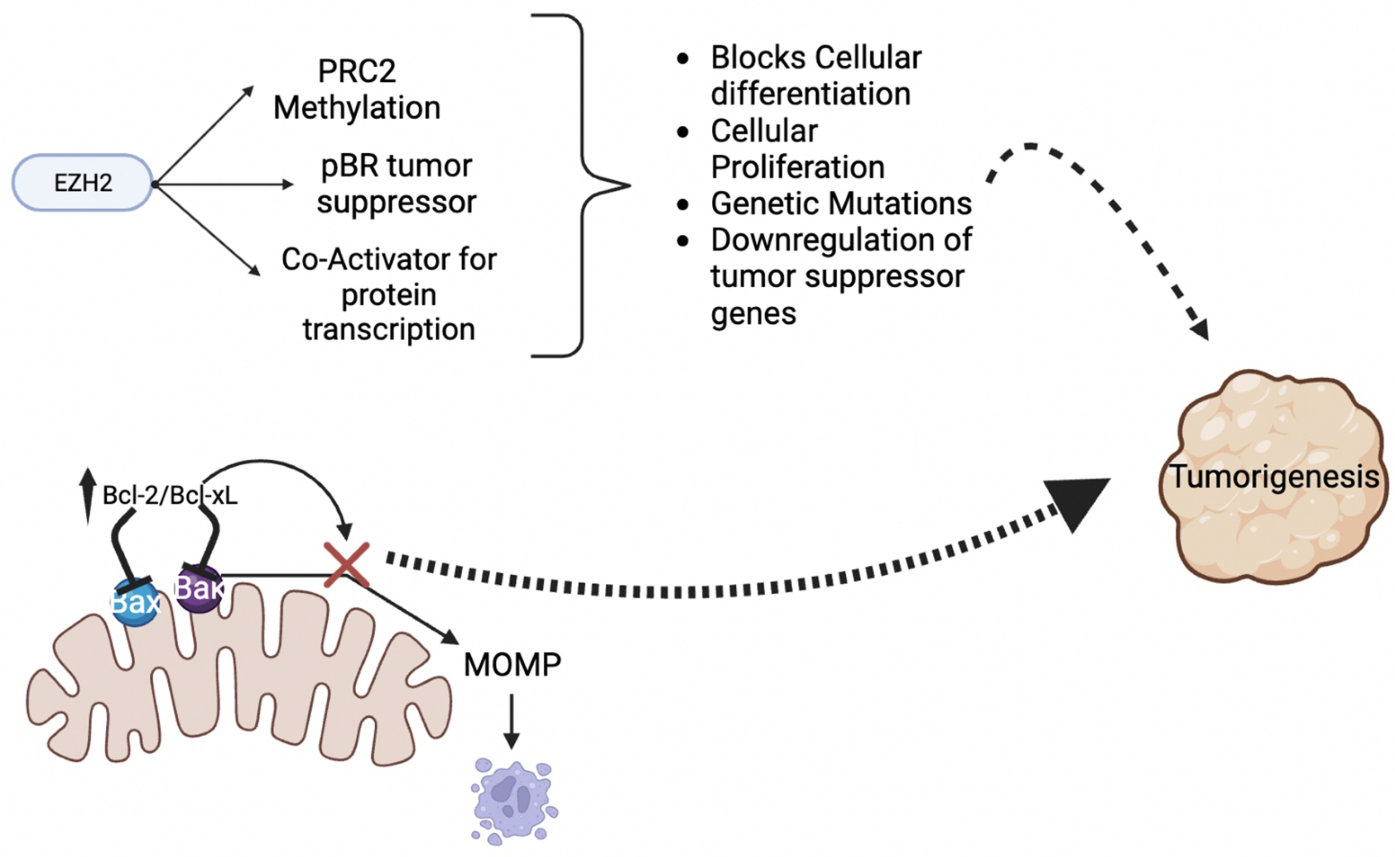
Intrinsic resistance mechanisms affecting tyrosine kinase inhibitor activity in RCC. Bcl-2: B cell lymphoma-2; Bcl-xL: B cell lymphoma-extra large; EZH2: enhancer of zeste homolog 2; PRC2: polycomb repressive complex 2; RCC: renal cell carcinoma.

B cell lymphoma-2 (Bcl-2) and B cell lymphoma-extra large (Bcl-xL) are proteins within the Bcl-2 family and play a key role in inhibiting apoptosis. These are known to be upregulated in many cancers, contributing to proliferation and metastasis^[90]^. There is limited evidence that overexpression of these proteins may confer intrinsic resistance to TKIs and other antineoplastic agents in RCC via inhibition of apoptosis, though further study is needed^[91]^. Figure 3 demonstrates known intrinsic mechanisms of resistance to TKIs in RCC.

## OVERCOMING TKI RESISTANCE IN RCC

Effective mitigation of TKI resistance plays a vital role in improving outcomes for patients with advanced RCC. Undoubtedly, this is a significant challenge due to the diverse range of mechanisms through which RCC can develop resistance. However, the multitude of mechanisms also offers numerous avenues for intervention, enabling the targeting of various processes to minimize resistance. Table 1 summarizes the many resistance mechanisms herein described, as well as potential approaches for overcoming these mechanisms.

**Table 1:** Acquired and intrinsic mechanisms of tyrosine kinase inhibitor resistance in RCC

**Process**	**Mechanism**	**TKIs affected**	**Potential therapies**	**Ref.**
Alternative proangiogenic pathways	Multiple; tumor hypoxia induces upregulation of interleukins, c-Met/HGF, angiopoietins, and growth factors	Sunitinib, pazopanib, sorafenib, axitinib, tivozanib	HIF-2α inhibitors; anti-IL-6 and IL-8 antibodies; TKIs against Ang 1 and 2, c-Met, and FGFR	[29-43,92-104]
Increased pericyte coverage	Increased tumor endothelial stability and production of VEGF	Sunitinib, pazopanib, sorafenib, axitinib, tivozanib	Unknown	[44-48]
Lysosomal sequestration	Drug sequestered; subtherapeutic intracellular concentrations; ineffective inhibition of receptor tyrosine kinases	Sunitinib	Alkalizing agents; drugs against lysosomal membrane proteins	[49-53,55,108]
Efflux pumps	Subtherapeutic intracellular concentrations	Sunitinib known, but other TKIs possible	P-GP inhibitors	[54,55,109-113]
Bone marrow-derived cell recruitment	Bone marrow-derived cells accumulate around tumor; immunomodulatory effects including suppression of cytotoxic T cells	Sunitinib, pazopanib, sorafenib, axitinib, tivozanib	Immune checkpoint inhibitors to rejuvenate immune response in the tumor microenvironment	[56-62]
Tumor-associated fibroblasts	Overactive fibroblasts secrete interleukins, hepatocyte growth factor, and more in tumor microenvironment; increased extracellular matrix deposition hampers drug penetration and supports cell adhesion	Unknown, but all TKIs possible	Histone deacetylase inhibitors	[63-67,105,107]
Epithelial-mesenchymal transition	Tumor hypoxia drives epithelial cells to acquire features such as increased motility, invasiveness, and resistance to cell death	Sunitinib, pazopanib, sorafenib, axitinib, tivozanib	Drugs with activity against EMT in combination with antineoplastic agents	[20,68-72,105,106]
Pharmacologic Factors	Single nucleotide polymorphisms in genes related to sunitinib metabolism lead to decreased concentrations of active metabolite	Sunitinib	Unknown	[3,74-80]
miRNAs	Silencing of tumor suppressor genes via destruction or decreased expression of mRNA	Sunitinib	Unknown	[81-86]
Methylation of tumor suppressor genes	EZH2 overexpression leads to aberrant methylation of tumor suppressor genes, rendering them inactive	All antineoplastic and targeted drugs	EZH2 inhibitors; hypomethylating agents	[87-89,114]
Inhibition of apoptosis	Bcl-2 and Bcl-xL overexpression inhibits tumor cell apoptosis	All antineoplastic and targeted drugs	Bcl-2 inhibitors	[90,91,115]

Bcl-2: B cell lymphoma-2; Bcl-xL: B cell lymphoma-extra large; EMT: epithelial-mesenchymal transition; EZH2: Enhancer of zeste homolog 2; FGFR: fibroblast growth factors receptor; HGF: hepatocyte growth factor; IL-6: interleukin-6; IL-8: interleukin-8; miRNAs: MicroRNAs; P-GP: P-glycoprotein; RCC: renal cell carcinoma; TKIs: tyrosine kinase inhibitors; VEGF: vascular endothelial growth factor.

One strategy is to target processes upstream from VEGF/VEGFR, such as HIF-2α. In hypoxic conditions, HIF-2α promotes the expression of multiple hypoxia-inducible genes, including VEGF and PDGF-BB. As previously discussed, in VHL syndrome, the negative regulatory protein pVHL is lost, leading to the accumulation of HIF-2α and excessive angiogenesis^[24]^. HIF-2α inhibitors act by inhibiting the dimerization of HIF-2α and its partner protein, ARNT1, thereby inhibiting HIF-2α mediated transcription^[92]^. The HIF-2α inhibitor, belzutifan, was approved by the FDA for the treatment of germline VHL-mutated RCC after a phase 2 trial demonstrated promising efficacy in this population^[93]^. Other studies examining belzutifan in sporadic (non-VHL associated) RCC are also encouraging^[94]^. In a phase 1 expansion cohort, belzutifan in combination with nivolumab was administered to patients with advanced ccRCC, and patients with therapeutic exposure to the drug had a median PFS of 10.0 months compared to just 4.7 months for patients with subtherapeutic exposure^[95]^. An ongoing trial evaluating the combination of belzutifan and cabozantinib is currently underway^[96]^.

Directly targeting the alternative proangiogenic pathways upregulated during resistance development may also be effective. IL-6 and IL-8, both upregulated in the setting of tissue hypoxia following effective antiangiogenic therapy, are prime examples^[47]^. These cytokines can induce a cascade of immunologic changes that promote angiogenesis. Interestingly, co-administration of IL-8 neutralizing antibody has shown promise in re-sensitizing xenograft tumors to sunitinib treatment^[97]^. Administration of the anti-IL-6 antibody, tocilizumab, given in combination with interferon, has also slowed RCC xenograft proliferation^[29]^. The Ang/Tie signaling pathway, which augments the VEGFR pathway and promotes blood vessel stabilization, survival, and maturation, can be directly targeted with the TKI trebananib^[98]^. In xenograft mouse models, treatment with trebananib in combination with sunitinib slowed tumor progression compared to treatment with sunitinib plus control^[36]^. However, other results have been less promising. A phase 2 trial examined the efficacy of trebananib with or without continued anti-VEGF therapy in RCC patients who had previously progressed on anti-VEGF therapy and observed poor outcomes in both treatment arms^[99]^. Targeting c-Met in cells that have become resistant to TKI’s against VEGF may also be effective. Studies have shown that the growth of sunitinib-resistant tumors can be slowed with a combination of sunitinib and a c-Met inhibitor^[39]^. Likewise, Zhou *et al.* found that treatment with cabozantinib, a TKI against VEGFR and MET, can rescue acquired sunitinib resistance in xenograft mouse models^[100]^. Finally, fibroblast growth factor (FGF) overexpression, specifically FGF2, has been associated with a poor RCC prognosis and contributes to TKI resistance through suppression of antiangiogenic activity^[42,101]^. There are several multitargeted TKIs that inhibit FGFR, such as lenvatinib, but these drugs are themselves susceptible to resistance^[102]^. *In vitro* studies have suggested that combining FGFR inhibitors with other TKIs such as PI3K inhibitors may help mitigate this resistance and inhibit RCC cell metabolic activity^[103]^. PI3K inhibitors have also demonstrated strong *in vitro* anti-tumor activity when co-administered with sunitinib^[104]^.

Targeting proangiogenic changes in the tumor microenvironment may be useful in combatting TKI resistance. The process of EMT, which promotes resistance to therapy and metastases, is associated with a host of metabolic changes. Although there are no direct inhibitors to EMT, multiple metabolism-inhibiting drugs have shown indirect activity against EMT changes through various complex biochemical mechanisms^[105]^. There are multiple ongoing clinical trials examining the combination of these drugs with antineoplastics agents, although none of these trials include RCC patients^[106]^. Additionally, TAFs may serve as a target for overcoming resistance. Histone deacetylase inhibitors offer a promising approach to diminish the activation of TAFs and eradicate their infiltration within the tumor stroma, as demonstrated by a study in patients with relapsed or refractory lymphoma or multiple myeloma^[105,107]^.

Lysosomal sequestration of TKIs is another potentially reversible mechanism of resistance. Combination therapies incorporating alkalizing treatments have been explored, creating a toxic environment that counteracts the sequestration process^[51]^. Drugs targeting various lysosomal membrane proteins have shown promise in destabilizing the lysosomal membrane, leading to membrane permeabilization^[55]^. Interestingly, Gotink *et al.* showed that sunitinib efficacy in RCC cells that had developed resistance via lysosomal sequestration can be restored by culturing resistant cells with sunitinib-free media^[108]^. This supports lysosomal sequestration as an acquired, transient, and reversible resistance mechanism.

Additionally, RCC has been shown to overexpress the ABC transporter P-glycoprotein (P-GP) in response to hypoxia from antiangiogenic therapy. P-GP is predominantly found in the cell membrane and exports drugs, contributing to multi-drug resistance^[109,110]^. P-GP is also commonly found in enterocytes and contributes to resistance via drug export into the GI lumen, limiting drug oral bioavailability^[111]^. Several studies have examined the co-administration of inhibitors to P-GP with anticancer drugs in various malignancies, but this strategy has been limited by severe toxicity and unwanted side effects^[112]^. Despite evidence of *in vitro* anti-tumor activity, there are currently no P-GP inhibitors approved for cancer treatment^[113]^.

Intrinsic mechanisms may also be targeted to overcome resistance. EZH2 overexpression in RCC contributes to hypermethylation, and ultimately inactivation, of tumor suppressor genes. The efficacy of EZH2 inhibitors and hypomethylating agents is currently not defined in many solid tumors, including RCC, but investigative studies are underway^[114]^. Antiapoptotic proteins such as Bcl-2 and Bcl-xL also confer intrinsic resistance. There is limited evidence regarding the use of Bcl-2 inhibitors in RCC. However, one preclinical study has demonstrated a potential synergetic effect of the Bcl-2 inhibitor, venetoclax, when given sequentially prior to sunitinib^[115]^.

In recent years, ICIs targeting programmed cell death protein 1 (PD-1), programmed cell death ligand 1 (PD-L1), and cytotoxic T lymphocyte-associated antigen 4 (CTLA-4) have emerged as a cornerstone in the treatment of advanced RCC. These therapies block interactions between immune checkpoint proteins, thereby triggering immune activation against various cancers^[116]^. ICIs are frequently combined with TKIs to leverage their synergistic effects resulting from complementary mechanisms of action. Notably, all TKI resistance mechanisms share a common thread of promoting angiogenesis. Proangiogenic molecules like VEGF impede both the innate and adaptive immune systems by hindering precursor cell differentiation, upregulating PD-1, PD-L1, and CTLA-4 on immune cells, and recruiting MDSCs^[117]^. ICI therapy rejuvenates the immune response, counteracting these immunosuppressive effects and mitigating resistance.

## CONCLUSION

TKIs have significantly improved the outcomes of patients with advanced RCC by targeting key pathways involved in cancer proliferation, survival, and metastasis. However, treatment with VEGF-targeted TKIs is almost always characterized by the eventual development of resistance and subsequent disease progression. Mitigating drug resistance is challenging due to the diverse mechanisms underlying TKI resistance, including upregulation of alternative proangiogenic pathways, EMT, efflux pumps reducing intracellular drug concentrations, lysosomal sequestration, alterations in the tumor microenvironment, and genetic factors.

Understanding these mechanisms is crucial for the development of innovative therapeutic approaches to overcome TKI resistance. Notably, the combination of TKIs with other agents, particularly ICIs, has made significant strides towards accomplishing this aim, solidifying ICIs as a cornerstone in the treatment of advanced RCC. Additionally, strategies to reverse or inhibit specific resistance mechanisms hold the potential to restore TKI efficacy. The continued exploration of combination therapies, comprehensive understanding of the tumor microenvironment, and identification of additional genetic biomarkers will pave the way for more effective treatments, ultimately improving outcomes for patients with advanced RCC.

## DECLARATIONS

### Copyright

© The Author(s) 2023.
